# High Rates of Homologous Recombination in the Mite Endosymbiont and Opportunistic Human Pathogen *Orientia tsutsugamushi*

**DOI:** 10.1371/journal.pntd.0000752

**Published:** 2010-07-20

**Authors:** Piengchan Sonthayanon, Sharon J. Peacock, Wirongrong Chierakul, Vanaporn Wuthiekanun, Stuart D. Blacksell, Mathew T. G. Holden, Stephen D. Bentley, Edward J. Feil, Nicholas P. J. Day

**Affiliations:** 1 Department of Clinical Tropical Medicine, Faculty of Tropical Medicine, Mahidol University, Bangkok, Thailand; 2 Mahidol-Oxford Tropical Medicine Research Unit, Faculty of Tropical Medicine, Mahidol University, Bangkok, Thailand; 3 Department of Medicine, University of Cambridge, Cambridge, United Kingdom; 4 The Wellcome Trust Sanger Institute, Hinxton, United Kingdom; 5 Department of Biology and Biochemistry, University of Bath, Bath, United Kingdom; 6 Centre for Tropical Medicine, Nuffield Department of Clinical Medicine, University of Oxford, Oxford, United Kingdom; University of Texas Medical Branch, United States of America

## Abstract

*Orientia tsutsugamushi* is an intracellular α-proteobacterium which resides in trombiculid mites, and is the causative agent of scrub typhus in East Asia. The genome sequence of this species has revealed an unprecedented number of repeat sequences, most notably of the genes encoding the conjugative properties of a type IV secretion system (T4SS). Although this observation is consistent with frequent intragenomic recombination, the extent of homologous recombination (gene conversion) in this species is unknown. To address this question, and to provide a protocol for the epidemiological surveillance of this important pathogen, we have developed a multilocus sequence typing (MLST) scheme based on 7 housekeeping genes (*gpsA*, *mdh*, *nrdB*, *nuoF*, *ppdK*, *sucD*, *sucB*). We applied this scheme to the two published genomes, and to DNA extracted from blood taken from 84 Thai scrub typhus patients, from 20 cultured Thai patient isolates, 1 Australian patient sample, and from 3 cultured type strains. These data demonstrated that the *O. tsutsugamushi* population was both highly diverse [Simpson's index (95% CI) = 0.95 (0.92–0.98)], and highly recombinogenic. These results are surprising given the intracellular life-style of this species, but are broadly consistent with results obtained for *Wolbachia*, which is an α-proteobacterial reproductive parasite of arthropods. We also compared the MLST data with *ompA* sequence data and noted low levels of consistency and much higher discrimination by MLST. Finally, twenty-five percent of patients in this study were simultaneously infected with multiple sequence types, suggesting multiple infection caused by either multiple mite bites, or multiple strains co-existing within individual mites.

## Author Summary

Scrub typhus, the rickettsial infectious disease caused by the obligate intracellular bacterium *Orientia tsutsugamushi*, is endemic across the Asia Pacific region. The bacterium is transmitted by the bite of larval stages of trombiculid mites (“chiggers”; *Leptotrombidium spp.*), which more typically feed on small rodents. Clinical features include fever, headache, myalgia, lymphadenopathy and an eschar at the site of the bite. Despite the importance of this pathogen, little is known of the population diversity or the role of homologous recombination in driving the microevolution of this species. Here, we describe the development and application of a multilocus sequence typing (MLST) scheme that can be applied directly to blood samples, and that was applied to 108 *O. tsutsugamushi* isolates. We found that this organism demonstrated a high rate of homologous recombination, a surprising finding given the intracellular life-style of this species. We also found that 25% of patients in our study were simultaneously infected with multiple sequence types, suggesting multiple infection caused by either multiple mite bites, or multiple strains co-existing within individual mites.

## Introduction

Scrub typhus is a zoonotic disease endemic in Southeast Asia caused by *Orientia tsutsugamushi*, a Gram-negative obligate intracellular coccobacillus. The number of new cases in East Asia has been estimated at approximately one million per year [1]. It is transmitted by the bite of larval stages of trombiculid mites (“Chiggers”; *Leptotrombidium spp.*), which more typically feed on small rodents. The disease commonly presents as an acute febrile illness within 7–10 days of being bitten. The clinical features include fever, headache, myalgia, lymphadenopathy and an eschar at the site of the bite. Disease severity and manifestations vary widely from asymptomatic to fatal, and show marked geographical differences, with reported fatality rates in the pre-antibiotic era ranging from 3% in Taiwan to 40% in Japan [2]. It is not known whether these geographical differences reflect genetic variation in the bacteria, the host, or both. Strains of *O. tsutsugamushi* are typically distinguished serologically on the basis of the 56 kDa-outer membrane protein encoded by *ompA*, which is known to be highly polymorphic within the natural population.

Despite the importance of this pathogen, little is known of the population diversity or the role of homologous recombination in driving the microevolution of this species. This question is relevant for the development of markers aimed at epidemiological surveillance, but is also of evolutionary interest given the unusual mode of molecular evolution and distinctive intracellular niche of this species. The genome of *O. tsutsugamushi* strain Boryong reveals a massive proliferation of repeated non-functional genes, including 359 copies of the conjugative transfer (*tra*) components of a type IV secretion system (T4SS), and >400 transposases [3]. These duplications may facilitate extensive intragenomic rearrangement and possibly homologous recombination, although direct population-based evidence for this is currently lacking.

Obligate intracellular bacteria are generally considered unlikely to undergo high rates of homologous recombination as strict vertical (transovarial) transmission from mother to offspring will lead to co-evolution of host and symbiont, and will restrict the opportunities for different lineages to meet, and hence recombine. This picture, which largely stems from extensive studies on the aphid symbiont *Buchnera*, has recently been challenged by convincing evidence of high rates of homologous recombination and host promiscuity in the α-proteobacterial reproductive parasite *Wolbachia* [4]. Current evidence implicates horizontal transmission between hosts to explain the lack of host specificity among different *Wolbachia* strains, and to provide the opportunity for different lineages to recombine [5]. Furthermore, the high proliferation of IS elements in the *Wolbachia* genome coincides with high rates of intragenomic rearrangements [6].

Here we have examined the role of homologous recombination in shaping the population structure of *O. tsutsugamushi* through the development of a multilocus sequence typing (MLST) scheme that can be performed directly on DNA extracted from patient blood. MLST is a powerful tool for the study of bacterial evolution [7], global epidemiological surveillance (e.g. *Streptococcus pneumoniae*, *Neisseria meningitidis*) and for monitoring the emergence of resistant strains (e.g. methicillin-resistant *Staphylococcus aureus*) [8]. We applied our MLST scheme to an incident series of scrub typhus infections in North and Northeast Thailand. We estimated the rate of homologous recombination expressed as the ratio of the likelihood that a given nucleotide site will change by a recombinational replacement of the region spanning the site against the likelihood that the site will change by *de novo* mutation (r/m). Comparisons with the equivalent estimates in other species point to very high rates of homologous recombination in *O. tsutsugamushi*. We have also shown some evidence for local clonal expansion and mixed infection, and have compared our results with those based on the highly polymorphic outer membrane protein encoded by *ompA*, which is currently used to distinguish strains.

## Materials and Methods

### Ethical statement

This study was conducted according to the principles expressed in the Declaration of Helsinki. The study protocol was approved by the Ethics Committee of the Faculty of Tropical Medicine, Mahidol University, Thailand (Approval Number: MUTM 2006-053). This retrospective study used the leftover sample. The subsequent data were analyzed anonymously.

### Patients and bacterial strains

Eighty-four patients presenting to Udon Thani general hospital, Northeast Thailand between October 2000 and December 2001 with scrub typhus were identified using PCR, as previously described [9]. Five millilitres of blood was drawn on admission for molecular diagnostics. The study also included 20 strains isolated previously from patients in Udon Thani and Tak province (Northern Thailand) that were maintained in laboratory culture. The bacterial reference strain Kato, DNA of reference strains Gilliam, Karp and a patient DNA ‘Sido’ were obtained from the Australian Rickettsial Reference Laboratory, Geelong, Australia. DNA was extracted from admission blood samples and *in vitro* cell culture as previously described [10].

### Gene choice and primer design

The housekeeping gene candidates were selected from shotgun sequencing of *O. tsutsugamushi* strain UT 76 (Udon Thani, Thailand), which was conducted at the Wellcome Trust Sanger Institute, UK (ftp://ftp.ensembl.org/pub/traces/orientia_tsutsugamushi_ut76). Using the incomplete assembly, contiguous genes homologous to 19 orthologous housekeeping genes from 8 related rickettsial species (*Rickettsia typhi*, *R. conorii*, *R. prowazekii*, *R. felis*, *Ehrlichia ruminantium*, *Anaplasma marginale*, *Wolbachia pipientis* strain *w*Mel, *Bartonella henselae*) were identified using BLASTN [11] and annotated using Artemis software [12]. Seven housekeeping gene loci were selected: *gpsA*, *mdh*, *nrdB*, *nuoF*, *ppdK*, *sucD*, *and sucB*. Fourteen primer pairs from these loci were designed using PrimerSelect (DNASTAR Lasergene, USA) (Table 1).

**Table 1:** Housekeeping genes and primers used in the *O. tsutsugamushi* MLST scheme.

Gene	Gene name	Primer	1^st^ PCR	Product	Primer	2^nd^ PCR	Product	MLST fragment
			sequence (5′->3′)	size (bp)		sequence (5′->3′)	size (bp)	size (bp)
*gpsA*	glycerol-3-phosphate dehydrogenase	gpsA_F	TCAGCCCATACTCAAGAAATCA	572	gpsA_NF	TCAGCTGCATACTAATAAAAA	510	390
		gpsA_R	GCAAATGCCACAATTTCCTT		gpsA_NR	GATGCTTTACAGTTTTGACCA		
*mdh*	malate dehydrogenase	mdh_F	CCAAAGCAGTTGCTCAAGGT	608	mdh_NF	AAAGCATGGGTATTGGTAAA	512	348
		mdh_R	AGCTGCTGCTGGAGCATAAT		mdh_NR	TCCTCCATCTCTAGTTCTTTGT		
*nrdB*	ribonucleoside-diphosphate reductase	nrdB_F	TAAAGCATGGCACACTCAGC	595	nrdB_NF	AAATTCACTGGCTACCAGAA	500	384
	beta subunit	nrdB_R	CTGTTCTGTCCAAACTTCAGGA		nrdB_NR	TGTTTCATCTCTAACTGACCA		
*nuoF*	NADH dehydrogenase chain F	nuoF_F	ATCTGGTTCTATGGCAGTTGAC	645	nuoF_NF	AAAATCTGGCTTACGTGGT	520	360
		nuoF_R	CATTTTGCGCCTCTTCTGAGTA		nuoF_NR	GAGTATTGTCGGAACTACAGC		
*ppdK*	pyruvate, phospate dikinase precursor	ppdK_F	CAAAGGTGTAACACTTGCTCAGA	591	ppdK_NF	TACCTATACCGCATGGTTTT	528	396
		ppdK_R	TGGTGGTTCATCCATGATTTT		ppdK_NR	ACTGCTTGAATAGCTTGGTG		
*sucB*	dihydrolipoamide S-succinyltransferase	sucB_F	CAGCAAAAGAAAGATGTTCAGC	590	sucB_NF	ATTGGCACAACTAATCCAGA	537	411
		sucB_R	GGTTGCCAAAATGGTAGCAG		sucB_NR	GCATAAAATCAATCCTGAGAA		
*sucD*	succinyl-CA synthase alpha chain	sucD_F	ATGTTCCTCCAGCTTTTGCT	599	sucD_NF	TGAAGCTATTGATGCTGGTA	562	411
		sucD_R	TCCAGCGCTTTTTAATGCTT		sucD_NR	AGCGCTTTTTAATGCTTCTA		

### PCR amplification and DNA sequencing

*O. tsutsugamushi* DNA was amplified using nested PCR, as follows. The first PCR round contained 200 µM dNTP, 1× PCR buffer, 1.5 mM MgCl_2_, 0.05 unit of *Taq* DNA Polymerase (Promega, USA) and 5 µl extracted DNA (total volume 50 µl). The amplification profile for all loci with the exception of *gpsA* was as follows: 94°C for 4 minutes (1 cycle), followed by 35 cycles of 94°C for 30 sec, 55°C for 30 sec, 72°C for 30 sec and 1 cycle of 72°C for 5 minutes. An annealing temperature of 50°C was used for *gpsA*. Five µl of the first PCR product was then used in a second PCR amplification profile using a 50°C annealing temperature for *sucD*, *nrdB*, *sucB*, *nuoF*, *ppdK* and 45°C for *mdh* and *gpsA*. PCR product clean up using QIAquick PCR purification kit (QIAGEN, Germany) was followed by sequencing reactions in forward and reverse directions using the second PCR primer. The PCR sequencing methods used ABI PRISM® BigDyeTM Terminator Cycle Sequencing Kits with AmpliTaq DNA polymerase (FS enzyme) (Applied Biosystems, USA), following the protocols supplied by the manufacturer. The PCR sequencing product was precipitated and then resuspended in loading buffer and subjected to electrophoresis in an ABI 3730XL sequencer (Applied Biosystems, USA).

### MLST and data analysis

MLST was defined for 84 DNA samples that had been extracted from EDTA blood and shown previously to be positive by PCR for *O. tsutsugamushi*, 21 DNA samples extracted from *in vitro O. tsutsugamushi* isolates, 3 DNA samples extracted (Karp, Gilliam and Sido strain), and 2 whole genome sequences available from GenBank. Forward and reverse sequence traces for each locus were compared using SeqMan® II (DNASTAR Lasergene, USA). Allele numbers for each locus were assigned to each unique sequence in the order in which they were discovered, to give an allelic profile for each strain in the order *gpsA-mdh-nrdB-nuoF-ppdK-sucD-sucB*. Each allelic profile was assigned a sequence type (ST), again numbered sequentially as new allelic profiles were found. The allele and profile frequencies were analysed using the software START version 2. The diversity index (Simpson's index of diversity) was calculated as previously described [13], [14]. The genetic relatedness on the basis of allelic profile of all samples (patient samples and reference strains) was analysed and displayed using e-BURST (https://eburst.mlst.net).

The DNA sequences from all 7 loci were concatenated in the locus order used to define allelic profile. A neighbour-joining tree based on the concatenated sequences was constructed using MEGA version 4.0. An estimate of the ratio of recent recombination to mutation events (r/m) with clonal complexes was made by comparing the sequences of mismatched alleles in clonal founders and single locus variants [15]. Other tests for recombination were performed using the RDP suite of programs [16].

### *ompA* (56-kDa) gene typing

The entire 56- kDa protein gene (1.5 kb) of the 22 *in vitro* isolates used in this study has been sequenced and reported previously [17], [18]. Comparisons were made between the 56kDa gene sequence data and MLST using BioNumerics (Applied Maths, Belgium). Simpson's index of diversity was calculated for each of these datasets.

### Restriction enzyme analysis and cloning

To verify that the double nucleotide peaks seen on sequencing were due to multiple gene products from two or more alleles of polymorphic genes present in the patient sample (indicative of mixed infection with multiple strains of *O. tsutsugamushi*), restriction enzyme analysis and PCR cloning were performed.

For restriction enzyme (RE) analysis, an enzyme was chosen to cut or not cut the PCR product once at a polymorphic site. PCR products of locus *gpsA* from 2 strains (no. 37 and 70) were digested with *Dde*I (Promega, USA) and *Nco*I (NEB, England) respectively, as recommended by the manufacturer. The restriction enzyme pattern was analysed by gel electrophoresis.

For PCR cloning, the PCR products from locus *gpsA* of strain no. 37 and 70 were blunt-end cloned to pGEM®T easy vector and transformed into *E. coli* JM109. 10–20 white colonies were selected and DNA was extracted and characterized. The clones of size greater than the vector (approximately 500-bp insert size) were further digested with *Eco*RI enzyme (NEB, England) to excise the cloned fragment and the products examined by gel electrophoresis. Clones with inserts of around 500 bp were digested with *Dde*I and *Nco*I. Selected clones that gave differing RE patterns were further verified by PCR and sequencing of the insert. Sequence trace was examined using SeqMan®II.

## Results

### Nested PCR for amplification of *O. tsutsugamushi* DNA from whole blood

The *O. tsutsugamushi* MLST scheme was developed for direct application to clinical blood samples. Most of the samples (77%) used in the current study were EDTA blood samples from patients with scrub typhus, an approach necessitated by the difficulty of isolating this slow growing bacterium in cell culture. Although DNA extracted directly from patients' blood may contain low concentrations of bacterial DNA, it was possible using a nested PCR approach to produce amplicons in concentrations that were sufficient to sequence. In this study there were 3 patient samples (UT125, UT144, UT196) on which MLST was performed both on DNA extracted from the original blood sample and from the organism grown in cell culture. The sequence types from both sources were identical (data not shown). This demonstrates that the MLST sequence type can be determined directly from a patient blood sample when *in vitro* culture is not available.

A total of 108 DNA samples (24 isolates extracted from *in vitro* cell culture and 84 PCR positive EDTA samples) were amplified and sequenced at all seven loci (2700-bp in total for each strain). The expected size of the final PCR products and the length of each sequenced gene fragment are shown in Table 1. Eighty-seven of the 108 DNA samples analysed had clear sequence reads in both directions at all 7 loci (Table S1), but 21 DNA samples, all amplified directly from patient blood (25% of patient samples processed in this way), repeatedly showed double peaks at one or more nucleotide positions at one or more loci. This was not seen with any of the 24 DNA samples extracted from cultured isolates. The number of polymorphic sites at each ambiguous locus varied from 1 to 13, and the number of ambiguous loci per strain varied from 1 to 7. The polymorphic sites in some strains were also found in other strains at the same positions.

### Analysis of MLST data

Forty-nine sequence types (STs) were identified among the 89 samples (84 Thai patient samples, 1 Australian patient sample, 2 reference samples and 2 *in silico* genomes available from GenBank) for which the sequencing was unambiguous at all loci (Table S1). Of these, 24 STs were represented by only a single strain. The most common ST was ST29, which accounted for 17 strains (19.1%), followed by ST2 (n = 7), ST6 (n = 5), ST33 (n = 4), ST34 (n = 4), ST27 (n = 3), ST1, 9, 10, 13, 30 and 38 (n = 2). The number of alleles at each locus ranged from 18 to 23. There was a high degree of genetic diversity with a Simpson's index of diversity of 0.95 (95% CI 0.92–0.98). The ratio of non-synonymous to synonymous nucleotide changes (dN/dS) was calculated for all 7 gene loci and found to range from 0.05–0.26 (Table 2), indicating that the genes are evolving predominantly by purifying selection.

**Table 2:** The genetic variation in *O. tsutsugamushi* MLST allele of 89 strains.

Gene	Length of allele (bp)	No. of lleles	No. of variable sites	d_N_/d_S_ †
*gpsA*	390	24	47	0.2663
*mdh*	348	20	18	0.2269
*nrdB*	384	19	21	0
*nuoF*	360	25	36	0.121
*ppdK*	396	21	45	0.2378
*sucB*	411	20	19	0.2605
*sucD*	411	20	26	0.0517

**†:** d_N_/d_S_: The ratio of mean non-synonymous substitutions per non-synonymous site and mean synonymous substitutions per synonymous site.

**Figure 1:**
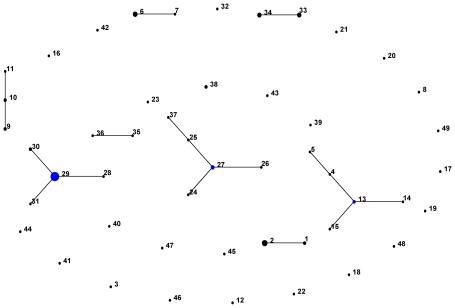
Population snapshot of 89 *O. tsutsugamushi* strains using e-BURST. The diagram demonstrates patterns of descent with lines linking the ancestral or founder strain (blue) and their descendents strain (black) for each clonal complex. The number represents the sequence type (ST) of each strain.

Using eBURST, 4 clonal complexes (CC) were identified (CC27, CC29, CC13 and CC10), as shown in the population snapshot of 89 strains in Figure 1. Clonal complexes were defined as sets of related STs that descended from the same founding genotype. Using a stringent definition of 6/7 shared alleles, CC27 contained ST 25, 26 and 24 as single locus variants (SLVs) and ST37 as a double locus variant (DLV; as an SLV of ST 25). Expanding the group definition to 5/7 loci in common resulted in the inclusion of four extra STs (35, 36, 38, 23) into CC27 (not shown). CC29 contained 3 SLVs (STs 30, 28 and ST31), CC13 contained 3 SLVs (STs 4, 14, 15) and 1 DLV (ST5), and CC10 contained 2 SLVs (ST9, 11). In addition there were four unconnected doublets at the 6/7 threshold, and 21 singletons. These links were entirely consistent with those defined using goeBURST, a recently developed optimized implementation of the BURST algorithm [19].

### Quantifying the rate of homologous recombination in *O. tsutsugamushi*

To understand the extent to which recombination has contributed to the diversification of this population compared with mutation, we estimated the ratio of recombination to mutation (r/m) at both the allelic and nucleotide level within clonal complexes by comparing the sequences of the non-identical alleles in all SLVs with their assigned clonal founders [20], [21]. Recombination is assumed to be the cause of multiple nucleotide changes (>1), while *de novo* mutation is assumed to be the cause if there is only a single nucleotide difference and if the resulting allele is not found elsewhere in the database. Of the 11 SLVs in 4 clonal complexes available for examination in our strain collection, only one genetic event was consistent with a point mutation by these criteria. The recombination to mutation ratio (r/m) per allele site is calculated from the number of alleles that were different in SLVs, and the per-site recombination to mutation was calculated from the overall number of nucleotide differences found in SLVs compare to the putative ancestral ST. The upper-bound ratio of recombination to mutation for *O. tsutsugamushi* in this population of 89 strains was estimated as 10∶1 at the allele level and 60∶1 at the nucleotide site level (r/m) (Table 3). These estimates are comparable to the freely recombining human pathogens *Neisseria meningitidis* and *Streptococcus pneumoniae* [22]. However, this estimate is based on only 11 SLVs, and a larger dataset is required in order to compute a more reliable estimate. Nevertheless, it is striking that 5/11 of the variant alleles in SLVs in the current data differ from the corresponding alleles in the founder for at least 8 nucleotide sites, (>1.5% sequence divergence). Thus even if many of the alleles differing by 2–4 nucleotides have emerged through point mutation and were misclassified as recombination events, the high diversity between these allelic comparisons points to a strong role for recombination.

**Table 3:** Variant alleles within the SLVs found in 4 clonal complexes.

Clonal complex	ST of clonal ancestor	ST of SLV	SLV frequency	Variant locus in SLV	Ancestral allele	SLV allele	No. of nucleotide differences*
27	27	25	1	*sucB*	6	2	2
	27	26	1	*sucB*	6	3	3
	27	24	1	*ppdK*	10	4	9
29	29	30	2	*sucB*	7	8	4
	29	28	1	*nrdB*	10	1	3
	29	31	1	*mdh*	9	12	1†
10	10	9	2	*nuoF*	3	1	11
	10	11	1	*sucB*	4	15	2
13	13	4	1	*gpsA*	4	2	8
	13	14	1	*sucD*	2	4	10
	13	15	1	*ppdK*	2	11	8

*When the number of nucleotide differences is more than one, this is assigned as having arisen through recombination.

**†:** Single nucleotide polymorphism of a novel allele not found elsewhere in the data set and therefore assigned as a point mutation.

**Figure 2:**
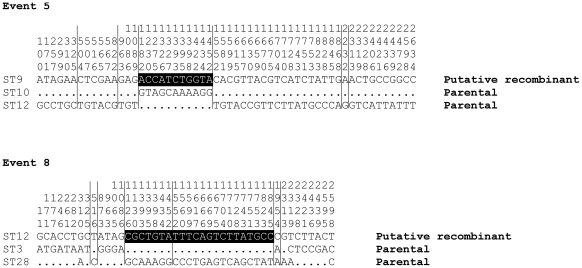
Mosaic structure in the 2 recombination events identified using the RDP suite of programs. The triple alignment of concatenated sequence of 3 STs (two parental STs and a putative recombinant) in the order *gpsA-mdh-nrdB-nuoF-ppdK-sucB-sucD*. Only variable sites within the concatenated alignment are shown. Allele borders are shown as vertical lines. Recombinant regions are shown as white on black. The vertical numbers on each variable site refer to position on sequence. The dot (.) represents the same nucleotide as shown in the first line sequence.

In order to find further evidence for recombination we used the RDP suite of programs. Six tests for recombination were employed on the concatenated sequences: Geneconv, Bootscan, Max Chi, Chimaera, SiScan and 3Seq. Together, these tests detected 85 recombination signals corresponding to 16 unique events. Eight of these recombination events were supported by at least 3 tests (P<0.05) (Table 4). Approximately half of the recombination breakpoints detected by these tests corresponded to gene borders, which suggests a role for both intra- and inter-genic recombination. We visually inspected the sequence trace of breakpoints detected by these tests, which confirmed striking mosaicism, and two examples (recombination events 5 and 8) are shown in Figure 2.

**Table 4:** The recombination events found in this population study using 6 recombination tests.

Event	Begin	End	Recombinant	Minor Parent	Major Parent	GECO	BTSC	MACH	CHIM	SISC	3Seq
1	**385**	**2272**	ST16	ST11	ST13	✗	✓	✗	✗	✗	✓
2	**385**	**2272**	ST14	ST4	Unknown	✗	✓	✓	✗	✓	✓
3	**18**	1608	ST18	ST3	ST34	✗	✗	✓	✓	✓	✓
4	**1497**	2168	ST38	Unknown	ST35	✓	✓	✓	✓	✓	✓
5	**1136**	**1502**	ST9	ST12	ST10	✓	✓	✗	✗	✓	✓
6	1261	**1875**	ST41	ST34	ST22	✗	✓	✓	✓	✓	✓
7	1846	431	ST7	ST5	ST47	✗	✓	✓	✓	✗	✓
8	**1106**	**1899**	ST12	ST3	ST28	✗	✗	✓	✓	✗	✓
9	1206	1614	ST22	Unknown	ST45	✗	✓	✓	✓	✓	✓
10	1208	1638	ST47	ST45	ST40	✗	✗	✗	✗	✓	✗
11	**18**	317	ST24	ST39	ST37	✗	✗	✗	✗	✗	✓
12	2153	2691	ST39	ST10	Unknown	✗	✗	✗	✗	✓	✗
13	**385**	**1509**	ST28	ST15	Unknown	✗	✗	✗	✗	✗	✓
14	1529	1821	ST7	Unknown	ST17	✗	✗	✗	✗	✓	✗
15	1510	1930	ST15	ST17	ST13	✗	✗	✗	✗	✗	✓
16	1507	**1875**	ST41	ST20	ST8	✗	✗	✗	✗	✗	✓

Begin and End are the breakpoint settings. ‘Recombinant’ is the putative recombinant which has sequence closely related to major parent and minor parent. ‘Minor parent’ is a ST that has sequence related to that from which sequences in the proposed recombinant region may have been derived. ‘Major parent’ is a ST that has sequence closely related to that from which the greater part of daughter's sequence (recombinant) may have been derived. Only one example of each parent and recombinant ST is shown for each event. Bold numbers are breakpoint at or near gene borders.

P-value of each tests are indicated as ✓: P<0.05 and ✗: Not Significant.

(GECO = GeneConv; BTSC = Bootscan; MACH = MaxChi squared; Chim = Chimaera; SISC = SiScan; 3Seq = 3Seq).

### Phylogenetic analysis and cluster definition

**Figure 3:**
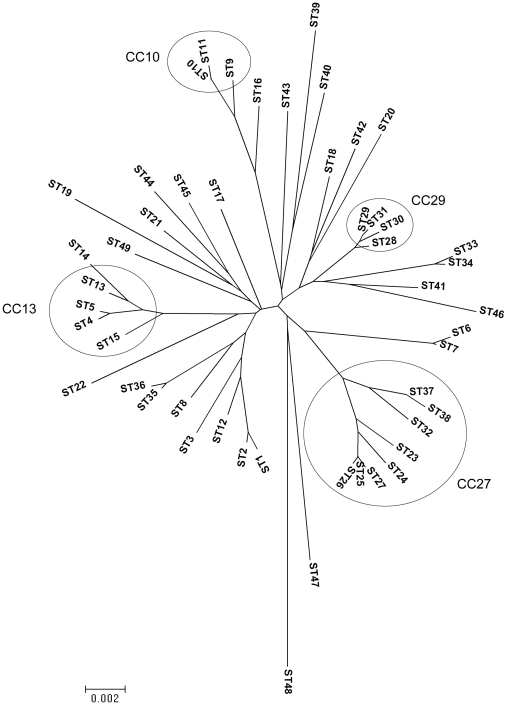
The phylogenetic analysis of 89 *O. tsutsugamushi* strains. A neighbour-joining tree was constructed on concatenated sequences of 87 strains from this study and 2 *in silico* strains obtained from GenBank. The circles indicate STs within each clonal complex (CC).

We examined the phylogeny of 49 STs including 47 STs from this study and the 2 sequenced strains Boryong and Ikeda from GenBank (accession no. AM494475 and AP008981, respectively), by using MEGA v 4.1 to construct a neighbour-joining tree (Figure 3). Although the boostrap values were generally very poor (not shown), the tree was broadly consistent with the clusters delineated by eBURST based on a group definition of 5/7 alleles in common. However, there were exceptions; STs 35 and 36 were excluded from CC27 by the tree (indicating recombination events between diverged parents), whereas ST32 was included in this group by the tree but excluded by eBURST at 5/7 loci (indicating mutational events at multiple loci).

### MLST and *ompA* (56-kDa) gene typing

We compared the discrimination provided by the 56-kDa gene sequence to the MLST data by comparing data for a set of 22 isolates using both methods. The 56-kDa gene sequence resolved the 22 isolates into 3 putative antigenic types (Gilliam, Karp, and TA716), based on comparisons to relevant reference sequences (Table 5). The 56-kDa gene sequence data were generally poorly congruent to the MLST data at both the ST level (31.4%) and the concatenated sequence level (18.1%). The MLST data resolved 15 STs, corresponding to a Simpson's Index of Diversity of 0.95 (0.91–0.99) compared to 0.48 (0.30–0.66) for the 56-kDa data. This data indicated that MLST has higher discrimination power than 56-kDa typing.

**Table 5:** Comparison of MLST data and putative antigenic type for 22 strains demonstrates that individual antigenic types contain numerous STs.

				MLST					56-kDa gene typing†	
Strains	*gpsA*	*mdh*	*nrdB*	*nuoF*	*ppdK*	*sucB*	*sucD*	*STs*	Putative antigenic type^1^	% identity^2^
UT076	1	1	1	1	1	1	1	1	Karp	93.1
UT167	1	1	1	1	1	3	1	2	Karp	93.2
UT316	1	1	1	1	1	3	1	2	Karp	93.2
UT332	1	1	1	1	1	3	1	2	Karp	93.2
UT150	2	2	1	2	2	2	2	4	Karp	93.2
UT169	2	2	1	2	2	2	3	5	Karp	95.7
FPW2031	2	4	3	4	4	1	5	6	Karp	94.7
UT213	2	4	3	4	4	1	5	6	Karp	95.3
UT221	2	4	3	4	4	1	5	6	Karp	95.4
UT219	2	4	3	4	4	1	5	6	Karp	95.5
UT395	2	4	3	4	4	2	5	7	Karp	95.4
UT176	3	3	2	3	3	4	4	10	Karp	94.6
UT177	3	3	2	3	3	4	4	10	Karp	94.4
UT336	4	2	1	2	2	2	2	13	Karp	96.1
UT418	6	6	5	5	6	2	2	19	Karp	93.6
UT144	11	9	10	10	7	7	10	29	Gilliam	91.4
UT196	11	9	10	10	7	7	10	29	Gilliam	91.4
UT125	11	12	10	10	7	7	10	31	Gilliam	91.4
UT329	12	10	9	11	7	3	11	34	Gilliam	91.8
FPW1038	15	13	13	16	15	13	13	40	TA716	95.9
FPW2016	16	1	14	17	7	14	14	41	Gilliam	88.9
FPW2049	17	1	15	18	7	4	15	42	Gilliam	89.8

**†:** Typing data based on the entire 56-kDa gene sequence, published previously in [17].

1 Type based on % identity to reference strains.

2 % identity compare with the relevant reference strain.

### Multiple genotypes in DNA sample from patients

**Figure 4:**
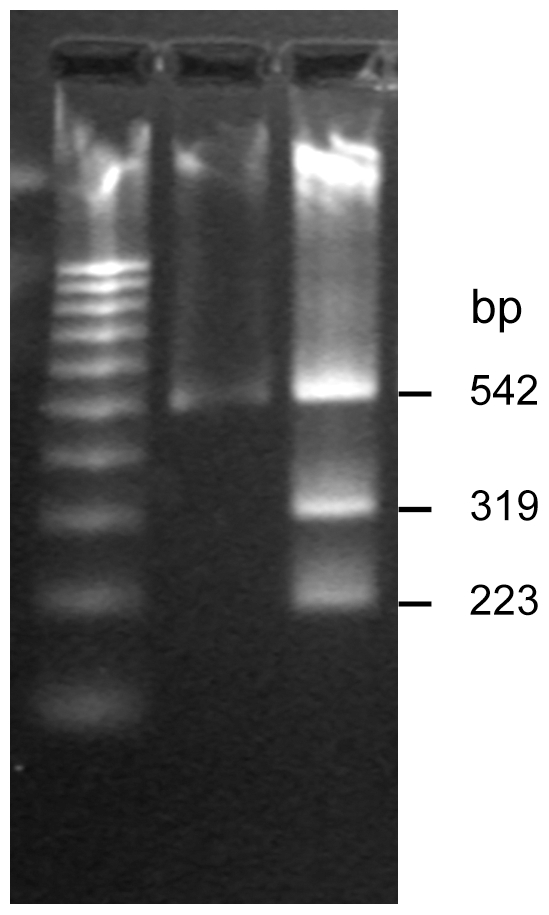
The restriction enzyme analysis on a predicted multiple infection *O. tsutsugamushi* strain. The DNA pattern on *O. tsutsugamushi* strain no. 37 undigested PCR product (1) and products after digestion with *Dde* I enzyme (2). M represents a 100 bp-DNA ladder marker.

**Figure 5:**
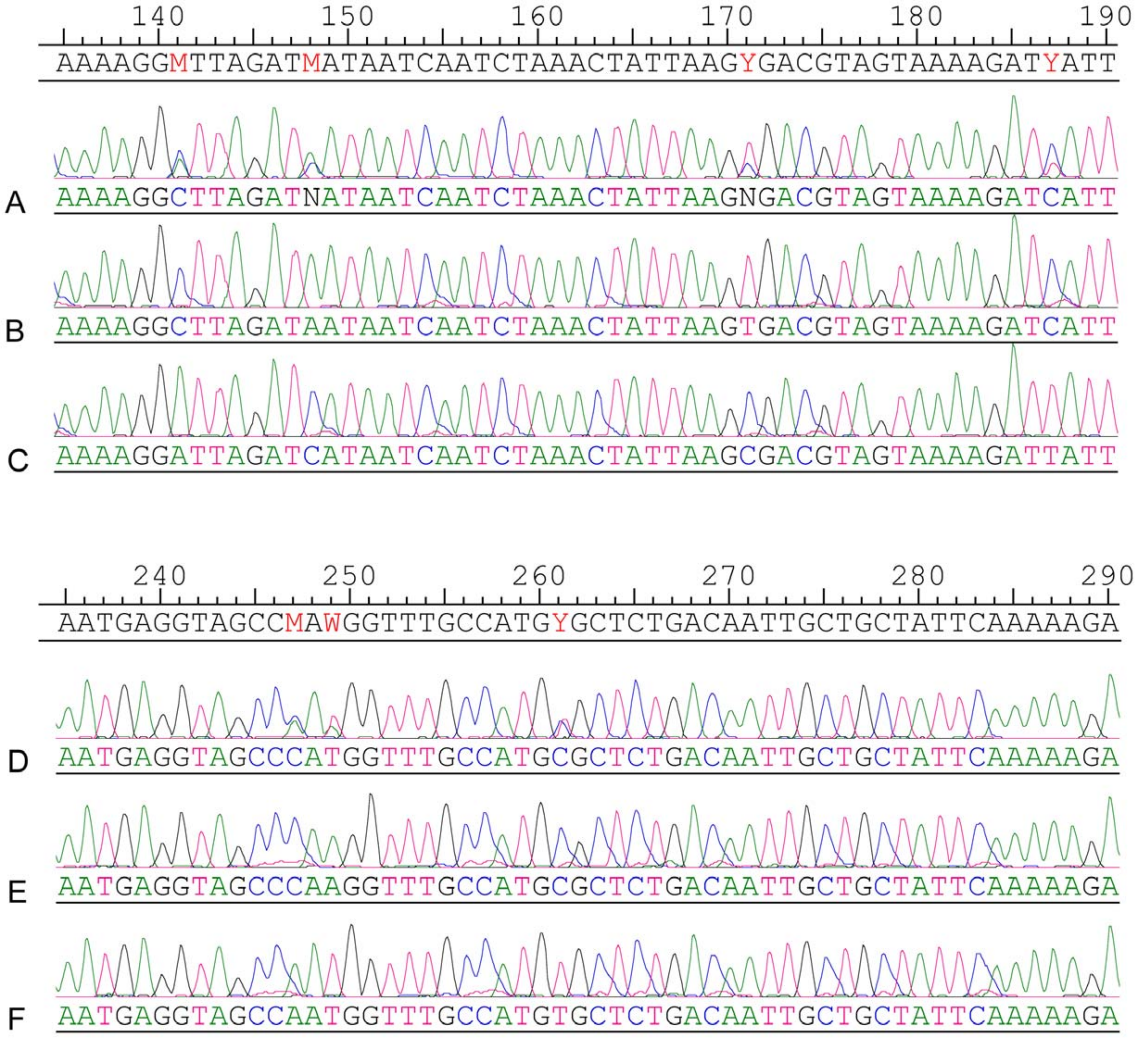
The sequence chromatogram comparison of two multiple infection strains and their PCR clone. Top alignment, strain no. 37 - A represents partial sequence trace of the PCR product of locus *gpsA* and B & C represent sequence from clones selected following PCR cloning of this amplicon. Bottom alignment, strain no. 70 - D represents partial sequence trace of the PCR product of locus *gpsA* and E & F represent sequence from clones selected from PCR cloning of this amplicon.

We repeatedly observed double peaks in the chromatograms from a number of DNA samples extracted directly from blood. For example, the 542 bp *gpsA* PCR product from strain no. 37 demonstrated a multiple (C/A) peak at position 223 and strain no. 70 demonstrated a multiple (T/C) peak at position 261. In order to check whether this resulted from a mixed infection we digested the PCR product with *Dde*I which was predicted to cut one of the two putative PCR products at a single polymorphic site [*Dde* I site (C^223▾^TNAG)]. Electrophoresis post digestion revealed 3 bands, two of the predicted size following *Dde* I digestion (at 319, 223 bp), and one representing an undigested product (at 542 bp) (Figure 4). Although this was consistent with the presence of two bacterial genotypes in the original patient blood sample, it is also possible that the three bands simply reflected partial digestion. We therefore cloned and sequenced *gpsA* amplicons from these samples to further evaluate the basis of the double peaks. The sequence of cloned amplicons resolved the double peaks by demonstrating the presence of either one or other nucleotide at these sites, confirming the presence of more than one PCR product in the original blood sample (Figure 5 A–F). We infer from this that the results are consistent with mixed infection in 25% of human samples tested, which can either be explained by mixed strains in mites or multiple bites of a single human by mono-infected mites. We also note that in many cases where multiple strains were recovered from a single blood sample, that strains tended to be more similar to each other than to other strains in the study. This may reflect the adaptation of particular bacterial genotypes to specific mite genotypes, although a more extensive dataset is needed from both human and mite hosts to examine this possibility more thoroughly.

## Discussion

Here we describe a new MLST scheme that was developed for *O. tsutsugamushi* and discuss evidence concerning the rates of recombination and mixed infection in the human host. Shotgun cloning and sequencing of a Thai *O. tsutsugamushi* isolate (UT 76 strain) greatly facilitated gene choice and the design of primers, and the genes have been confirmed to be ubiquitous within the *O. tsutsugamushi* population and are likely to be predominantly under neutral selection. We therefore argue that the MLST genes proposed here fulfill all the criteria suggested for large-scale typing [7] and form a representative sample of the core *O. tsutsugamushi* genome.

We noted a large number of STs and high allelic diversity at all loci within the 89 *O. tsutsugamushi* strains characterized by MLST. The population of *O. tsutsugamushi* is thus very diverse (Simpson's index 0.95), with a high number of STs per strain (49 STs in 89 strains, 0.55 STs per strain). Estimation of the relative contributions of recombination and mutation to the emergence of variant alleles provides insight into the way a bacterial population is diversifying. This disease-causing *O. tsutsugamushi* population showed high r/m ratios at both the allelic (10∶1) and nucleotide level (60∶1), suggesting that the diversification of natural populations of *O. tsutsugamushi* is predominantly characterized by recombination rather than mutation and is comparable with other human pathogens known to recombine freely: *Neisseria meningitidis* (3.6∶1 and 100∶1); *Streptococcus pneumoniae* (8.9∶1 and 61∶1) and *Helicobacter pylori* (6.7∶1 and 76∶1). Our estimated r/m ratio does not take account of the patient population who were putatively infected by more than one strain of *O. tsutsugamushi*. It is not possible to resolve the STs in these cases but we have no reason to think that inclusion of these data would lead to a reduction in this ratio.

The genome sequence of *O. tsutsugamushi* shows characteristics that are consistent with high rates of recombination [3], [23]. Sixty percent of functional genes have been reported to be involved in replication, recombination and repair processes [24]. In addition, the Boryong sequence strain has a massive proliferation of mobile elements and repeat sequences. Horizontal gene transfer probably occurs more readily due to the high number of mobile elements. The constant shuffling of DNA may in turn have ecological implications, such as facilitating host-adaptation.

Comparison of MLST to a single locus typing method (based on the gene for the immunodominant surface expressed 56-kDa protein) showed low congruence between these two methods. Simpson's index, which is used to assess the discriminative ability of typing methods, was higher for MLST (0.95) than for the single locus typing method (0.48). However, the number of isolates used in this assessment was low (n = 22), and further investigation is needed to accurately assess the relative abilities of the two methods. In general, typing that relies on antigenic gene variation, which is subject to diversifying selection from the immune response, is less able to reveal the underlying population genetic structure, although such approaches may be useful for characterising local outbreaks.

The use of DNA extracted from patient blood enabled us to detect the presence of multiple infecting genotypes in a single patient sample. The finding that approximately 25% of patients had multiple MLST genotypes in their blood suggests that either the patient had been bitten by multiple mites harboring different strains, or that several strains of *O. tsutsugamushi* coexist in single mites. This second hypothesis is supported by the detection of multiple antigenic strains of *O. tsutsugamushi* in both naturally infected and laboratory-reared chigger mites (*Leptotrombidium spp.*) [25]. This implies that different strains of *O. tsutsugamushi* may commonly coexist in the same place at the same time, providing an opportunity for genetic exchange to occur and variation to arise. Recombination between different strains of *O. tsutsugamushi* could either occur in the mite, or in the rodent reservoir which may become infected on multiple, independent occasions. Further studies are now needed to investigate the molecular epidemiology of *O. tsutsugamushi* harboured by mites and rodents.

## Supporting Information

Table S1 Strain details and MLST data for 89 *O. tsutsugamushi* strains included in this study.
(0.14 MB DOC)
