# Supplementary material for: High Rates of Homologous Recombination in the Mite Endosymbiont and Opportunistic Human Pathogen Orientia tsutsugamushi
Source: PLoS Negl Trop Dis. 2010 Jul 20;4(7):e752. doi: 10.1371/journal.pntd.0000752 (PMC2907413; doi:10.1371/journal.pntd.0000752)
Supplement: Table S1 — Strain details and MLST data for 89 O. tsutsugamushi strains included in this study. (0.14 MB DOC) [file pntd.0000752.s001.doc]

# Table S1: Strain details and MLST data for 89 *O. tsutsugamushi* strains included in this study.

| **Strain** | **country** | **region** | **ST** | **allelic number at each locus** | | | | | | |
| --- | --- | --- | --- | --- | --- | --- | --- | --- | --- | --- |
|  |  |  |  | ***gpsA*** | ***mdh*** | ***nrdB*** | ***nuoF*** | ***ppdK*** | ***sucB*** | ***sucD*** |
| UT76 | Thailand | Udon Thani | 1 | 1 | 1 | 1 | 1 | 1 | 1 | 1 |
| 652 | Thailand | Udon Thani | 1 | 1 | 1 | 1 | 1 | 1 | 1 | 1 |
| UT167 | Thailand | Udon Thani | 2 | 1 | 1 | 1 | 1 | 1 | 3 | 1 |
| UT332 | Thailand | Udon Thani | 2 | 1 | 1 | 1 | 1 | 1 | 3 | 1 |
| 494 | Thailand | Udon Thani | 2 | 1 | 1 | 1 | 1 | 1 | 3 | 1 |
| 682 | Thailand | Udon Thani | 2 | 1 | 1 | 1 | 1 | 1 | 3 | 1 |
| UT316 | Thailand | Udon Thani | 2 | 1 | 1 | 1 | 1 | 1 | 3 | 1 |
| 55 | Thailand | Udon Thani | 2 | 1 | 1 | 1 | 1 | 1 | 3 | 1 |
| 80 | Thailand | Udon Thani | 2 | 1 | 1 | 1 | 1 | 1 | 3 | 1 |
| 217 | Thailand | Udon Thani | 3 | 2 | 1 | 11 | 1 | 1 | 7 | 16 |
| UT150 | Thailand | Udon Thani | 4 | 2 | 2 | 1 | 2 | 2 | 2 | 2 |
| UT169 | Thailand | Udon Thani | 5 | 2 | 2 | 1 | 2 | 2 | 2 | 3 |
| UT213 | Thailand | Udon Thani | 6 | 2 | 4 | 3 | 4 | 4 | 1 | 5 |
| UT219 | Thailand | Udon Thani | 6 | 2 | 4 | 3 | 4 | 4 | 1 | 5 |
| UT221 | Thailand | Udon Thani | 6 | 2 | 4 | 3 | 4 | 4 | 1 | 5 |
| FPW2031 | Thailand | Tak | 6 | 2 | 4 | 3 | 4 | 4 | 1 | 5 |
| 41 | Thailand | Udon Thani | 6 | 2 | 4 | 3 | 4 | 4 | 1 | 5 |
| UT395 | Thailand | Udon Thani | 7 | 2 | 4 | 3 | 4 | 4 | 2 | 5 |
| UT497 | Thailand | Udon Thani | 8 | 2 | 11 | 10 | 15 | 14 | 12 | 5 |
| 555 | Thailand | Udon Thani | 9 | 3 | 3 | 2 | 1 | 3 | 4 | 4 |
| 30 | Thailand | Udon Thani | 9 | 3 | 3 | 2 | 1 | 3 | 4 | 4 |
| UT176 | Thailand | Udon Thani | 10 | 3 | 3 | 2 | 3 | 3 | 4 | 4 |
| UT177 | Thailand | Udon Thani | 10 | 3 | 3 | 2 | 3 | 3 | 4 | 4 |
| 75 | Thailand | Udon Thani | 11 | 3 | 3 | 2 | 3 | 3 | 15 | 4 |
| 679 | Thailand | Udon Thani | 12 | 4 | 1 | 1 | 1 | 1 | 2 | 2 |
| UT336 | Thailand | Udon Thani | 13 | 4 | 2 | 1 | 2 | 2 | 2 | 2 |
| 83 | Thailand | Udon Thani | 13 | 4 | 2 | 1 | 2 | 2 | 2 | 2 |
| 239 | Thailand | Udon Thani | 14 | 4 | 2 | 1 | 2 | 2 | 2 | 4 |
| 523 | Thailand | Udon Thani | 15 | 4 | 2 | 1 | 2 | 11 | 2 | 2 |
| 228 | Thailand | Udon Thani | 16 | 4 | 3 | 2 | 3 | 3 | 15 | 2 |
| 433 | Thailand | Udon Thani | 17 | 4 | 9 | 11 | 13 | 11 | 10 | 12 |
| UT340 | Thailand | Udon Thani | 18 | 5 | 5 | 4 | 1 | 5 | 3 | 6 |
| UT418 | Thailand | Udon Thani | 19 | 6 | 6 | 5 | 5 | 6 | 2 | 2 |
| Kato^*^ | Japan | Niigata | 20 | 7 | 1 | 6 | 6 | 7 | 5 | 7 |
| 681 | Thailand | Udon Thani | 21 | 8 | 6 | 7 | 7 | 8 | 2 | 8 |
| 514 | Thailand | Udon Thani | 22 | 9 | 7 | 1 | 8 | 9 | 2 | 9 |
| 397 | Thailand | Udon Thani | 23 | 10 | 1 | 8 | 12 | 10 | 6 | 5 |
| **Strain** | **country** | **region** | **ST** | **allelic number at each locus** | | | | | | |
|  |  |  |  | ***gpsA*** | ***mdh*** | ***nrdB*** | ***nuoF*** | ***ppdK*** | ***sucB*** | ***sucD*** |
| 56 | Thailand | Udon Thani | 24 | 10 | 8 | 8 | 9 | 4 | 6 | 5 |
| 48 | Thailand | Udon Thani | 25 | 10 | 8 | 8 | 9 | 10 | 2 | 5 |
| 404 | Thailand | Udon Thani | 26 | 10 | 8 | 8 | 9 | 10 | 3 | 5 |
| 520 | Thailand | Udon Thani | 27 | 10 | 8 | 8 | 9 | 10 | 6 | 5 |
| 297 | Thailand | Udon Thani | 27 | 10 | 8 | 8 | 9 | 10 | 6 | 5 |
| 37 | Thailand | Udon Thani | 27 | 10 | 8 | 8 | 9 | 10 | 6 | 5 |
| 532 | Thailand | Udon Thani | 28 | 11 | 9 | 1 | 10 | 7 | 7 | 10 |
| 586 | Thailand | Udon Thani | 29 | 11 | 9 | 10 | 10 | 7 | 7 | 10 |
| 591 | Thailand | Udon Thani | 29 | 11 | 9 | 10 | 10 | 7 | 7 | 10 |
| 622 | Thailand | Udon Thani | 29 | 11 | 9 | 10 | 10 | 7 | 7 | 10 |
| 658 | Thailand | Udon Thani | 29 | 11 | 9 | 10 | 10 | 7 | 7 | 10 |
| 403 | Thailand | Udon Thani | 29 | 11 | 9 | 10 | 10 | 7 | 7 | 10 |
| 418 | Thailand | Udon Thani | 29 | 11 | 9 | 10 | 10 | 7 | 7 | 10 |
| 420 | Thailand | Udon Thani | 29 | 11 | 9 | 10 | 10 | 7 | 7 | 10 |
| 466 | Thailand | Udon Thani | 29 | 11 | 9 | 10 | 10 | 7 | 7 | 10 |
| UT144 | Thailand | Udon Thani | 29 | 11 | 9 | 10 | 10 | 7 | 7 | 10 |
| UT196 | Thailand | Udon Thani | 29 | 11 | 9 | 10 | 10 | 7 | 7 | 10 |
| UT210 | Thailand | Udon Thani | 29 | 11 | 9 | 10 | 10 | 7 | 7 | 10 |
| 298 | Thailand | Udon Thani | 29 | 11 | 9 | 10 | 10 | 7 | 7 | 10 |
| 323 | Thailand | Udon Thani | 29 | 11 | 9 | 10 | 10 | 7 | 7 | 10 |
| 118 | Thailand | Udon Thani | 29 | 11 | 9 | 10 | 10 | 7 | 7 | 10 |
| 221 | Thailand | Udon Thani | 29 | 11 | 9 | 10 | 10 | 7 | 7 | 10 |
| 49 | Thailand | Udon Thani | 29 | 11 | 9 | 10 | 10 | 7 | 7 | 10 |
| 63 | Thailand | Udon Thani | 29 | 11 | 9 | 10 | 10 | 7 | 7 | 10 |
| 552 | Thailand | Udon Thani | 30 | 11 | 9 | 10 | 10 | 7 | 8 | 10 |
| 676 | Thailand | Udon Thani | 30 | 11 | 9 | 10 | 10 | 7 | 8 | 10 |
| UT125 | Thailand | Udon Thani | 31 | 11 | 12 | 10 | 10 | 7 | 7 | 10 |
| 367 | Thailand | Udon Thani | 32 | 12 | 8 | 9 | 9 | 10 | 2 | 1 |
| 537 | Thailand | Udon Thani | 33 | 12 | 10 | 9 | 11 | 7 | 1 | 11 |
| 440 | Thailand | Udon Thani | 33 | 12 | 10 | 9 | 11 | 7 | 1 | 11 |
| 250 | Thailand | Udon Thani | 33 | 12 | 10 | 9 | 11 | 7 | 1 | 11 |
| 46 | Thailand | Udon Thani | 33 | 12 | 10 | 9 | 11 | 7 | 1 | 11 |
| 599 | Thailand | Udon Thani | 34 | 12 | 10 | 9 | 11 | 7 | 3 | 11 |
| UT329 | Thailand | Udon Thani | 34 | 12 | 10 | 9 | 11 | 7 | 3 | 11 |
| 257 | Thailand | Udon Thani | 34 | 12 | 10 | 9 | 11 | 7 | 3 | 11 |
| 293 | Thailand | Udon Thani | 34 | 12 | 10 | 9 | 11 | 7 | 3 | 11 |
| 697 | Thailand | Udon Thani | 35 | 13 | 1 | 8 | 9 | 1 | 2 | 5 |
| 78 | Thailand | Udon Thani | 36 | 13 | 2 | 8 | 9 | 1 | 2 | 5 |
| 698 | Thailand | Udon Thani | 37 | 13 | 8 | 8 | 9 | 10 | 2 | 5 |
| 571 | Thailand | Udon Thani | 38 | 13 | 8 | 8 | 9 | 12 | 9 | 5 |
| 655 | Thailand | Udon Thani | 38 | 13 | 8 | 8 | 9 | 12 | 9 | 5 |
| 478 | Thailand | Udon Thani | 39 | 14 | 11 | 12 | 14 | 13 | 11 | 4 |

| **Strain** | **country** | **region** | **ST** | **allelic number at each locus** | | | | | | |
| --- | --- | --- | --- | --- | --- | --- | --- | --- | --- | --- |
|  |  |  |  | ***gpsA*** | ***mdh*** | ***nrdB*** | ***nuoF*** | ***ppdK*** | ***sucB*** | ***sucD*** |
| FPW1038 | Thailand | Tak | 40 | 15 | 13 | 13 | 16 | 15 | 13 | 13 |
| FPW2016 | Thailand | Tak | 41 | 16 | 1 | 14 | 17 | 7 | 14 | 14 |
| FPW2049 | Thailand | Tak | 42 | 17 | 1 | 15 | 18 | 7 | 4 | 15 |
| 244 | Thailand | Udon Thani | 43 | 18 | 14 | 2 | 19 | 16 | 1 | 6 |
| 62 | Thailand | Udon Thani | 44 | 19 | 15 | 16 | 20 | 17 | 16 | 17 |
| Karp^*^ | New Guinea | - | 45 | 20 | 16 | 17 | 21 | 17 | 4 | 11 |
| Gilliam^*^ | Burma | - | 46 | 21 | 17 | 14 | 22 | 18 | 17 | 18 |
| Sido^**^ | Australia | Torres Strait island | 47 | 22 | 18 | 18 | 23 | 19 | 18 | 19 |
| Boryong^†^ | Korea | - | 48 | 23 | 19 | 19 | 24 | 20 | 19 | 20 |
| Ikeda^†^ | Japan | Niigata | 49 | 24 | 20 | 14 | 25 | 21 | 20 | 7 |

* Reference prototype and ** patients DNA from Australian Rickettsial Reference Laboratory

^†^ *in silico* from GenBank
